# Wild microbiomes of striped plateau lizards vary with reproductive season, sex, and body size

**DOI:** 10.1038/s41598-022-24518-6

**Published:** 2022-11-30

**Authors:** Marie E. Bunker, A. Elizabeth Arnold, Stacey L. Weiss

**Affiliations:** 1 Department of Biology, University of Puget Sound, Tacoma, WA USA; 2 School of Plant Sciences and Department of Ecology and Evolutionary Biology, The University of Arizona, Tucson, AZ USA

**Keywords:** Microbial ecology, Microbiome, Herpetology

## Abstract

Long-term studies of animal microbiomes under natural conditions are valuable for understanding the effects of host demographics and environmental factors on host-associated microbial communities, and how those effects interact and shift over time. We examined how the cloacal microbiome of wild *Sceloporus virgatus* (the striped plateau lizard) varies under natural conditions in a multi-year study. Cloacal swabs were collected from wild-caught lizards across their entire active season and over three years in southeastern Arizona, USA. Analyses of 16S rRNA data generated on the Illumina platform revealed that cloacal microbiomes of *S. virgatus* vary as a function of season, sex, body size, and reproductive state, and do so independently of one another. Briefly, microbial diversity was lowest in both sexes during the reproductive season, was higher in females than in males, and was lowest in females when they were vitellogenic, and microbiome composition varied across seasons, sexes, and sizes. The pattern of decreased diversity during reproductive periods with increased sociality is surprising, as studies in other systems often suggest that microbial diversity generally increases with sociality. The cloacal microbiome was not affected significantly by hibernation and was relatively stable from year to year. This study highlights the importance of long term, wide-scale microbiome studies for capturing accurate perspectives on microbiome diversity and composition in animals. It also serves as a warning for comparisons of microbiomes across species, as each may be under a different suite of selective pressures or exhibit short-term variation from external or innate factors, which may differ in a species-specific manner.

## Introduction

Ecological and evolutionary dynamics of macroscopic organisms are influenced by species interactions, and increasingly are informed by studies of their microbiomes^1^. However, even in controlled lab conditions, the microbiome/host relationship can be a complicated study system. Challenges are compounded under natural conditions, where variables such as life stage, movement, stress, or health conditions may interact with shifting environmental factors to impact the host-associated microbial community^2–5^. Perhaps not surprisingly, meaningful trends in microbiome structure across species and ecosystems have proved difficult to codify in many cases^6^. Studies on microbiomes of animals under wild conditions have found that they may vary as a function of factors such as host sex^7,8^, age^3,4,9^, reproductive state^10,11^, season^2,7,12^, diet^9,13,14^, and habitat^2,15,16^. These factors often are studied separately, but they can interact with one another in complex ways that make identification of causal drivers in microbiome shifts difficult to discern. For instance, microbiome variation between the sexes may change during reproduction in sparrows^7^, seasonal changes have been linked to a corresponding shift in diet in mice^11^ and some reptiles^12^, and seasonal changes only affect the microbiome of certain body regions in red squirrels^17^. Long-term, large-scale datasets are often difficult and time consuming to collect in these natural systems, but they are vital for parsing the impact of these nested or interacting influences on wild microbiomes.

The focus for many of these studies is on changes and fluctuations of the microbiome, but stability is also an important finding. Often researchers will try to identify a “core” microbiome that is resident in the host species regardless of demographics and resilient to change from external factors—a suite of microbes often referred to as a “common” core^18–20^. This is in contrast to considering the entire microbial community, which can include rare and transient taxa that are more affected by the factors described above. Often the core microbiome is defined by genetic function, rather than taxonomy, as these core microbes ostensibly have an important function in the host, making them more likely to persist across evolutionary time^19,20^. However, rare or transient taxa outside the persistent and abundant core can also have important impacts on host health and related factors^21–23^. These taxa can be included instead in a “temporal” core, defined by taxa that persist over time in a community, even if they are less prevalent than “common core” microbes^19^. Thus, utilizing various definitions of the core microbiome and tracking changes over time, life stages, and environmental shifts can provide complementary and mutually informative perspectives on host-associated microbes.

Here we present a multi-year study of the cloacal microbiome of *Sceloporus virgatus* (the striped plateau lizard), an oviparous spiny lizard that occurs in Mexico and southern Arizona and New Mexico, USA. The cloacal microbiome of *S. virgatu*s protects eggs from pathogenic fungi during incubation^24^ and thus is important in both the ecological and evolutionary dynamics of this species. Previous work has shown that the *S. virgatus* cloacal microbiome is similar to that of the lower intestine, but is distinct from that of the upper intestine, oviduct, and fecal pellets^25^. In the present study we examined how the cloacal microbiome varies in wild *S. virgatus* as a function of physiological variation—sex, size, and reproductive state in females—along a seasonal gradient. This gradient also is defined by reproduction, but encompasses both males and females in the (1) pre-reproductive season, in the early spring, prior to females becoming vitellogenic; (2) the reproductive season, which encompasses vitellogenic and gravid animals through egg laying; and (3) the post-reproductive season after egg laying. These seasons are characterized by ecological and behavioral shifts that have been shown to impact the microbiome^26^, and we predicted that the increase in copulatory and other social behaviors during the reproductive season will lead to changes in microbial diversity and composition. To place our inferences in a broader seasonal framework, we compared the microbiome in September and April, the two timepoints immediately preceding and following hibernation. Overall the aims of this study were (1) to identify how the microbiome of *S. virgatus* varies in diversity and composition as a function of these factors, both individually and in concert with one another, and (2) to test the prediction that given the importance of the cloacal microbiome to reproductive success, a “core” microbiome may exist for which functional traits would be especially important for future study.

## Methods

### Sample collection

We studied the cloacal microbiome of wild *S. virgatus* in Coronado National Forest near the Southwestern Research Station (SWRS) in Cochise County, AZ, USA. The study site, located at ca. 1740 m a.s.l., follows an intermittent creek surrounded by an oak-juniper mixed forest. In this area, *S. virgatus* are found on the ground, rocks, logs, and trees, and subsist on a diet of arthropods. Males and females have overlapping home ranges^27^. They typically mate in May (late spring and the beginning of the arid foresummer), lay eggs in early July (at the onset of the North American Monsoon and associated summer rains), and emerge as hatchlings toward the end of the monsoon season (August and September)^28^.

We began field sampling on 22 May 2017, capturing lizards and collecting cloacal swabs at 2-week intervals until animals went dormant for winter (10 October 2017). We resumed the following spring on 20 March 2018, and sampled until 24 July 2018. Additional samples were collected in May and June 2019. At each sampling interval, we swabbed at least 10 males and 10 females, except during the approach to winter dormancy when activity was dwindling.

Lizards were captured with a loop of fishing line at the end of a retractable fishing pole. The cloaca of each lizard was swabbed in the field by gently inserting a sterile swab (BD ESwab™) into the cloaca and slowly rotating it. Demographic information was collected from each individual (sex, snout-vent length (SVL), and female reproductive state (by abdominal palpation), and then each animal was toe-clipped for permanent identification and released. Some animals were re-sampled by chance throughout the study, but no effort was made to intentionally re-capture individuals. Overall, our sampling included 257 cloacal swabs from 209 individual lizards (Table 1). Control swabs, which sampled the air, researchers’ hands, and the lizards’ outer vents were collected at each sampling interval. Swabs were stored at − 80 °C until DNA could be extracted.

**Table 1:** Sampling of the cloacal microbiome of wild *S. virgatus* in three reproductive seasons, three years, and with regard to sex of sampled individuals.

Sex	Year	Reproductive season			
		Pre-reproductive	Reproductive	Post-reproductive	Mean SVL
Female	2017	–	16	38	63.9
	2018	29	29	5	
	2019	–	16	–	
Male	2017	–	21	26	57.7
	2018	23	27	6	
	2019	–	15	–	

Values are sample sizes and mean snout-vent length (SVL) for the two sexes. In 2017 and 2018, sampling occurred every two weeks throughout the sampling period. In 2019, sampling occurred at only two time points (late May and late June). —indicates no sampling. Table excludes 6 females for which the reproductive state did not match the reproductive season (as defined in *Statistical Analyses*); these 6 animals were only included in the reproductive state analysis, below.

All protocols were approved by the University of Puget Sound Institutional Animal Care and Use Committee (PS16002 and PS18002) and the Arizona Game and Fish Department (SP590934, SP616620, SP649069), and were carried out in accordance with relevant guidelines and regulations. Methods are reported here following recommendations in the ARRIVE guidelines (https://arriveguidelines.org).

### Sample processing and filtering

Total genomic DNA was extracted from swabs via the Qiagen DNEasy Blood and Tissue Kit (Qiagen). We used the manufacturer’s protocol for purification of total DNA from animal blood or cells, with the optional pre-treatment for gram-positive bacteria (i.e., incubation in lysis buffer). This extraction method may favor certain gram-positive bacteria, but it has been used successfully in previous work^7,24,25^. Methods for amplification via the polymerase chain reaction (PCR) and Illumina sequencing are described in Bunker et al.^24^, informed by Taylor et al.^3^. Briefly, Illumina libraries were prepared via a two-step approach. We performed PCR 1 in triplicate to maximize the amount of diversity captured, with primer pairs 515F and 806R to amplify the V4 region of the 16s rRNA. Replicates were pooled for each sample. PCR2 added unique barcode primers. Samples were then pooled according to qualitative DNA concentration. Pooled PCR2 libraries were sent to the University of Idaho Genomics and Bioinformatics Resource Core (GRBC) for sequencing on the Illumina MiSeq platform.

Paired end sequences were pre-processed by demultiplexing, with adapters and primers removed. Quality analysis for each sample was performed using FastQC^29^ and those results were consolidated using MultiQC^30^. Mean quality scores and length distribution for the whole dataset were inspected manually and used to determine a cutoff length of 270 bp for forward reads and 175 bp for reverse reads, from a total length of 291 bp. The dataset was processed in R v4.0.2 via the DADA2 pipeline, following https://benjjneb.github.io/dada2/tutorial.html. We used analyses of mock communities (BEI Resources, ATCC, Manassas, VA) to establish the remaining parameters for data processing (see Supplemental File 1). Samples were trimmed and filtered with a maximum expected error (EE) of 2. An average of 87.6% of reads were kept in all experimental samples after processing.

Taxonomic classification of amplicon sequence variants (ASVs) was performed through the assignTaxonomy function with the Silva database^31^ release 132. Potential contaminants were removed with the Decontam package^32^ with the “prevalence” method set with a threshold of 0.1. Control samples (n = 50), including field controls, extraction blanks, and PCR negatives were used with Decontam. Any ASV that had fewer than 10 reads across all samples was discarded. Read numbers were log-transformed to account for differences in read depth, based on analysis of the mock communities (Supplemental File 1). Once samples had been processed, the phyloseq package^33^ was used to organize and store data of different types for analysis.

Samples to be included in the final analyses then were narrowed further. Previous research on the cloacal microbiome of *S. virgatus* showed that fecal samples and cloacal swabs yield distinct communities, and cloacal swabs can sometimes be contaminated by feces if they are collected around the time of defecation^25^. In that case, the community is more similar to that of the transient fecal microbiome (which is not representative of the gut as a whole) rather than the resident cloacal microbiome, which is the intended target for this study. High relative abundance of the family *Lachnospiraceae* was identified as an indicator that cloacal swabs have been contaminated by fecal material, as the family makes up a large proportion of the community in fecal samples but is essentially absent from cloacal tissue^25^. Therefore, any samples for which > 5% reads were assigned to *Lachnospiraceae* were removed. (See Supplemental File 2 for further justification of the 5% cutoff.) Although we cannot confirm without further investigation that there are no fecal microbes in our remaining “clean” samples, this filtering is designed to minimize the contaminated samples in this analysis and focuses our findings on resident cloacal microbes. Data processing was then repeated in the same manner as above, using only the samples that passed this quality-control set and thus were included in the analysis.

### Statistical analyses

We calculated diversity (Shannon index) and richness (Observed) with the “estimate_richness function” from phyloseq. Faith’s phylogenetic diversity index values (PD) were calculated with the picante package^34^ based on phylogenetic trees created and optimized with phangorn^35,36^. The alignment (length 544 bp) was created with the DECIPHER package^37^. The same tree was used to generate pairwise weighted and unweighted UniFrac distances. Bray–Curtis distances between samples were calculated with the vegan package^38^. All plots were generated in ggplot2^39^.

We analyzed beta diversity metrics via permutational analysis of variance (PERMANOVA) using adonis2 in the vegan package^38^. We examined the assumption of equal dispersion via betadisper in vegan^38^. Because differences in dispersion between communities is informative, we report results of both tests in all cases. Principal coordinate analysis (PCoA) plots were generated for beta diversity metrics using the “ordinate” function in phyloseq^33^. We analyzed alpha diversity metrics via ANOVA with animal ID as a random factor. We examined test assumptions via diagnostic plots (residuals vs. fitted values and Q-Q plots). Shannon diversity values met assumptions of equal variance and normality. Richness and PD met these assumptions only after log transformation. For all models, we removed nonsignificant interaction terms and present here only the simplified models.

Using these approaches, we assessed inter-annual stability of the cloacal microbiome by comparing samples gathered in May and June of 2017, 2018, and 2019, and by comparing pre- and post-hibernation samples. We then combined data from all three years to ask how the microbiome varies with reproductive season (three biologically relevant periods during the lizard’s active season: pre-reproductive season, ranging from April 02–May 15; reproductive season, May 16–July 15; and post reproductive season, July 16–September 12), body size, and sex. We considered both the total recovered microbiome and the core microbiome. We defined a common core microbiome for the whole community, as well as separately for each categorical variable of interest (i.e., both sexes, and all three reproductive seasons), based on prevalence across samples, using the core function in the microbiome package^40^. The overall and reproductive-season common cores were defined as ASVs present in 50% of samples or greater, while the sex cores were defined by ASVs present in 40% of samples or greater, as there were no ASVs present in 50% of males. Both male and female lizards were included to define the core in each season, and animals from all seasons were included to define the core for each sex, as we found no interaction between these factors in other analyses. Additionally, we collapsed ASVs to the family level and defined a common family core for each variable (at 50% prevalence). We defined a temporal core microbiome based on ASVs that were present over time and across all the categorical variables investigated. This core comprises 121 ASVs that made up 92% of the community on average.

Finally, we examined how microbiomes varied as a function of reproductive state, considering only females. This included females that were classified as pre-reproductive, vitellogenic, gravid, and post-oviposition. Vitellogenic and gravid females were combined in the “reproductive season” category for other analyses.

### Ethical approval

The work was permitted by the University of Puget Sound Institutional Animal Care and Use Committee (PS16002 and PS18002) and the Arizona Game and Fish Department (SP590934, SP616620, SP649069).

## Results

After processing, the cloacal microbiome dataset from wild *S. virgatus* consisted of 4,369,996 reads representing 1892 ASVs. Of these, only 31 ASVs accounted for greater than 1.0% of the community on average. These included 12 classified as an unknown genus in *Enterobacteriaceae* (Proteobacteria), 11 classified as *Helicobacter* (*Helicobacteraceae*, Proteobacteria), and eight classified as *Izhakiella* (*Enterobacteriaceae*, Proteobacteria). The greatest average relative abundance of any one of these ASVs was 1.6% (an unknown *Enterobacteriaceae*), and the largest relative abundance of any one ASV within an individual was 11.1% (*Helicobacter*). Overall, 295 ASVs were assigned to *Enterobacteriaceae* (74.9% of total reads), 49 to *Helicobacteraceae* (22.7% of total reads), and 38 to *​​Enterococcaceae*, which only accounted for 0.5% of total reads. For comparison, 165 ASVs were assigned to *Bacteroidaceae* and 110 to *Tannerellaceae*, but those families only accounted for 0.4% and 0.2% of total reads, respectively. This indicates that a relatively small number of ASVs in the three most abundant families, particularly *Enterobacteriaceae* and *Helicobacteraceae,* have a disproportionately large relative abundance in this community.

### Long time-scale stability

**Figure 1:**
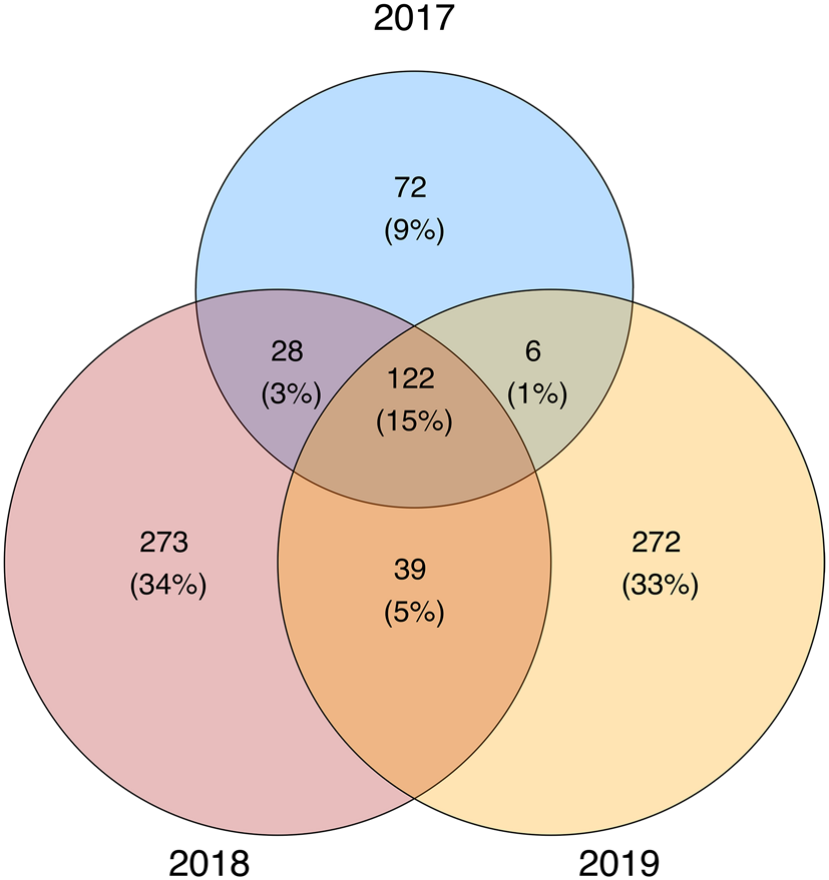
Overlap of ASVs recovered from *S. virgatus* cloacal microbiomes across three sampling years (2017, 2018, 2019) in May and June.

We did not detect differences via PERMANOVA among May and June samples collected over three years in terms of microbial community structure or composition using any distance measure (Bray–Curtis: p = 0.257, R^2^ = 0.03; weighted UniFrac: p = 0.230, R^2^ = 0.04; unweighted UniFrac: p = 0.082, R^2^ = 0.04) (Table S1). We also found no difference among years in diversity, richness, or phylogenetic diversity of the cloacal microbiome (ANOVA, Shannon: p = 0.157; Richness: p = 0.146; PD: p = 0.112; Table S2). Overall, 24% of ASVs were found across at least two years, and 15% of ASVs were found in all three years (Fig. 1). All but three of these shared ASVs were members of *Enterobacteriaceae* (95 ASVs) or *Helicobacteraceae* (24 ASVs).

Samples collected pre-hibernation (September) vs. immediately post-hibernation (April) also did not differ meaningfully from each other via PERMANOVA (Bray–Curtis: p = 0.082, R^2^ = 0.01; weighted UniFrac: p = 0.688, R^2^ = 0.01; unweighted UniFrac: p = 0.387, R^2^ = 0.02; Table S3). We also did not detect differences in any alpha diversity metric across the hibernation period in an ANOVA (Shannon: p = 0.505; Richness: p = 0.486; PD:, p = 0.735) (Table S4). This indicates remarkable resilience of the microbiome over long time scales. Therefore, we combined all three sampling years for the remaining analyses.

### Variation relative to reproductive season, sex, and size

**Figure 2:**
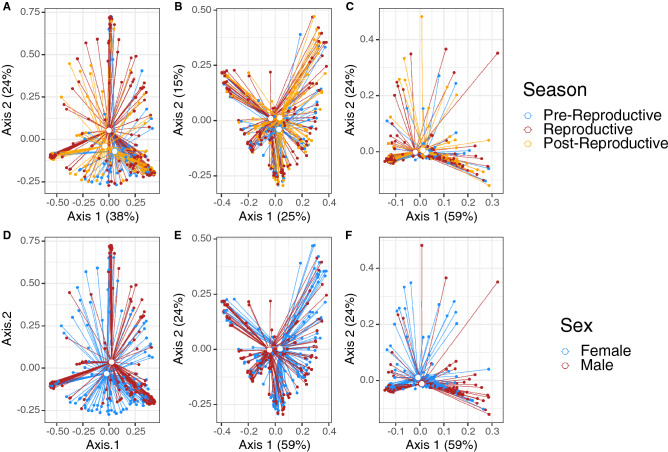
PCoA plots for all cloacal microbiomes, based on (**A**) Bray–Curtis, (**B**) unweighted UniFrac, and (**C**) weighted UniFrac distances. Open dots represent the centroids of each group. Solid dots represent each sample. Segments connect centroids to each sample. Colors represent either season of the active period (**A**–**C**) or sex (**D**–**F**).

The cloacal microbiome differed as a function of reproductive season (Fig. 2A, B), sex (Fig. 2D–F), and size (SVL), as evaluated by Bray–Curtis (season: p = 0.007, R^2^ = 0.02; sex: p = 0.032, R^2^ = 0.01; SVL: p = 0.014, R^2^ = 0.01) and unweighted UniFrac distances (season: p = 0.002, R^2^ = 0.02; sex: p = 0.005, R^2^ = 0.01; SVL: p = 0.001, R^2^ = 0.02) with a PERMANOVA (Table S5). Examination of weighted UniFrac distances by PERMANOVA showed that the microbiome only differed significantly in response to sex (p = 0.034, R^2^ = 0.01) (Fig. 2F) and SVL (p = 0.044, R^2^ = 0.01), though there was still a trend of separation for reproductive seasons (p = 0.071, R^2^ = 0.02, Fig. 2C, Table S5).

**Figure 3:**
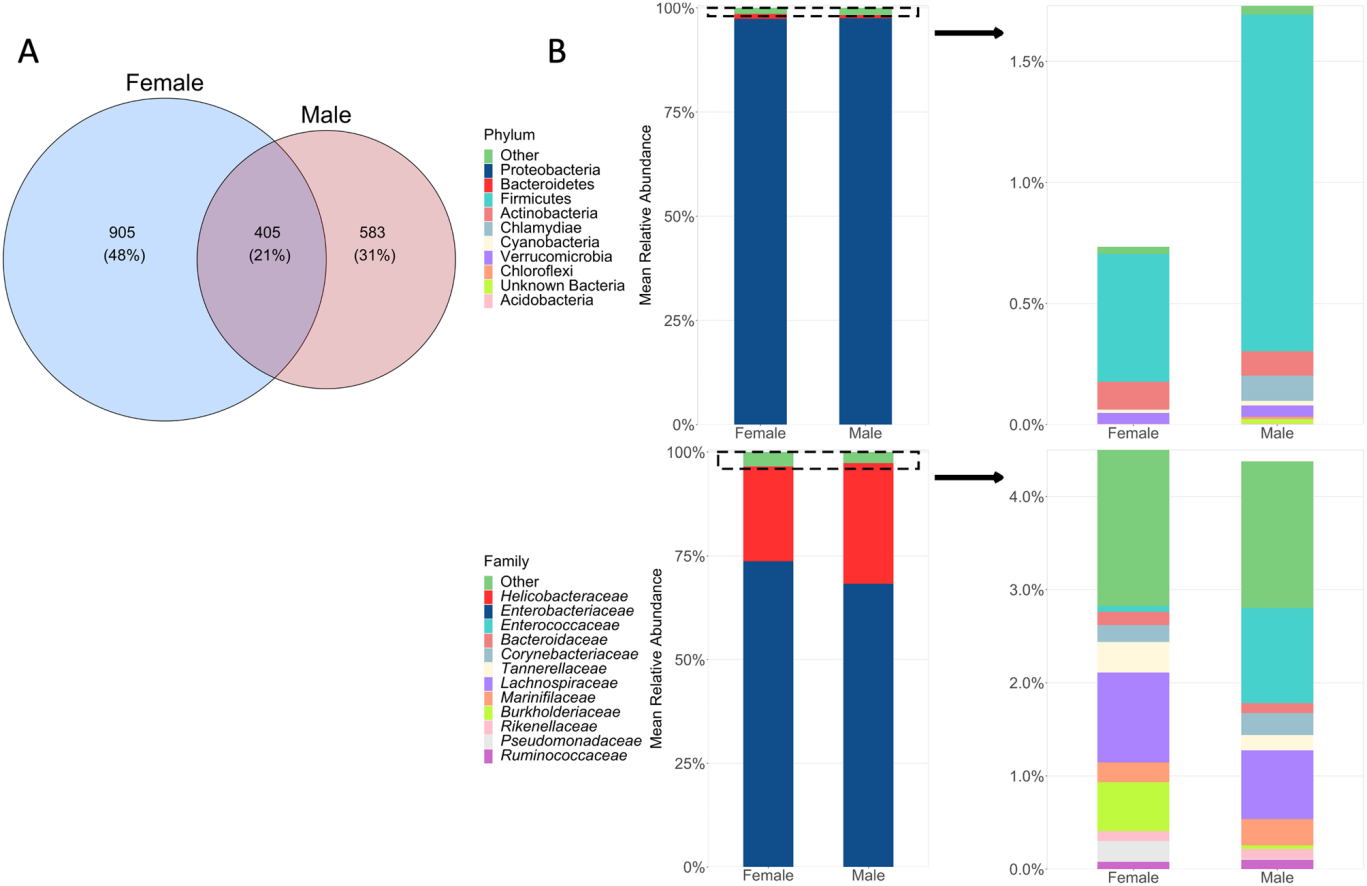
(**A**) Overlap of ASVs recovered from male and female *S. virgatus* cloacal microbiomes. (**B**) Percent composition of cloacal microbiomes in male and female *S. virgatus*. In the left-side plots, different colors of the bars represent the relative abundance of the top 2 most abundant phyla and families; the remaining taxa were combined into the “other” category. Within that category, the next 10 most abundant taxa are represented by different colors on the right-side plots, and the remaining taxa again grouped into the “Other” category. The y-axis of both panels indicates the average percent of total reads for each sex that each taxa represents.

Overall, 48% of ASVs in all samples were unique to females, with only 21% of ASVs shared across the sexes (Fig. 3A). Despite differences in diversity and composition at the ASV level, the cloacal microbiome of both sexes was dominated by *Enterobacteriaceae*, comprising an average of 73.7% ± 2.4 (SE) of the community in females and 68.3% ± 3.4 of the community in males (Fig. 3B). For both sexes, *Helicobacteraceae* was the next most abundant family (females: 22.8% ± 2.4; males: 29.1% ± 3.3), as well as the only other family comprising > 1% of the community on average. At the phylum level, Proteobacteria comprised over 97% of the community (females: 97.4% ± 0.5; males: 97.5% ± 0.6; Fig. 3B). Thus, significant differences in microbiome diversity and composition between sexes represented variation at lower taxonomic levels.

The dominance of *Enterobacteriaceae* persists across the lizards’ active season. The lowest diversity season (reproductive season) had the highest abundance of *Enterobacteriaceae* (76.7% ± 2.7 of reads) relative to the pre-reproductive season (63.2% ± 4.4) and post-reproductive season (67.7% ± 4.1). These changes correspond to a shift in abundance of *Helicobacteraceae,* which is lower during the reproductive season (20.6% ± 2.6) than during the pre-reproductive (31.5% ± 4.4) and post-reproductive (30.0% ± 4.1) seasons. The combined abundance between these two families was 95–98% across all three seasons.

**Figure 4:**
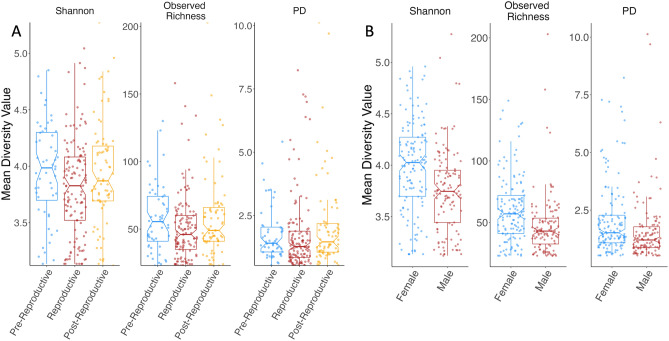
Diversity (Shannon index), Observed Richness, and Faith’s phylogenetic diversity (PD) of cloacal microbiomes (**A**) for *S. virgatus* over three reproductive seasons, and (**B**) for males and females across the entire active period (April through September). Boxes represent median and quartiles, while whiskers indicate 95% confidence intervals.

Shannon diversity and richness, but not phylogenetic diversity, differed significantly across the reproductive season (ANOVA, Shannon: p = 0.026, richness: p = 0.024, PD: p = 0.141; Fig. 4A). Reproductive animals consistently hosted the least diverse community, compared to pre- and post-reproductive animals. Shannon diversity, richness, and PD were significantly higher in females compared to males (ANOVA, Shannon: p < 0.001; richness: p < 0.001, PD: p = 0.001; Fig. 4B). Body size did not impact any alpha diversity metric significantly (ANOVA, Shannon: p = 0.484, richness: p = 0.472, PD: p = 0.218) (Table S6).

### Core microbiomes

Only nine ASVs were found in over 50% of samples, to be included in an overall common core (Table S7). Of these nine ASVs, seven belonged to the *Enterobacteriaceae* family, and two to *Helicobacteraceae.* Due to the low number of taxa in this common core, we then defined the core microbiome for each sex and reproductive season individually to compare them (Table S7). The female core included 68 ASVs, all of which belonged to either the *Enterobacteriaceae* or *Helicobacteraceae*. The male core only contained 25 ASVs. All ASVs present in the male core were also in the female core, which had an additional 43 ASVs unique to the female core. When the common core was defined at the family level, the female core consisted of *Enterobacteriaceae*, *Helicobacteraceae*, and *Corynebacteriaceae,* while the male core only contained *Enterobacteriaceae* and *Helicobacteraceae.*

The pre-reproductive, reproductive, and post-reproductive cores contained 33, 2, and 16 ASVs, respectively. The two ASVs present in the reproductive season core (both *Enterobacteriaceae*) were both found in the pre-reproductive core, but only one was maintained in the post-reproductive core. The pre- and post-reproductive cores shared six ASVs, including five *Helicobacteraceae* and one *Enterobacteriaceae*. At the family level, pre- and post-reproductive cores contained *Enterobacteriaceae*, *Helicobacteraceae*, and *Corynebacteriaceae*, and the breeding core only contained *Enterobacteriaceae* and *Helicobacteraceae*.

We next examined the relevance of reproductive season, sex, and SVL to the composition of the temporal core microbiome as defined in the Methods (which contained a sufficient richness of ASVs for statistical analysis; Table S7). All ASVs in the temporal core were present in at least 12 samples and in as many as 136 samples. When using Bray–Curtis distance, we found that the temporal core microbiome varied depending on all three factors, each of which was independently informative (season: p = 0.011, R^2^ = 0.02; sex: p = 0.032, R^2^ = 0.01; SVL: p = 0.015, R^2^ = 0.01). When using weighted UniFrac distance, the core communities only varied depending on SVL (p = 0.03, R^2^ = 0.02), with a trend of separation for sex (p = 0.066, R^2^ = 0.01) but not season (p = 0.264, R^2^ = 0.01). Using unweighted UniFrac distance, core communities varied in response to sex (p = 0.043, R^2^ = 0.01) and SVL (p = 0.006, R^2^ = 0.02), but not season (p = 0.104, R^2^ = 0.01) (Table S8). Although effect sizes are small for both the temporal core microbiome and the whole microbiome community, consistency in results suggests that this data set captures true biological variation.

### Female reproductive state

**Figure 5:**
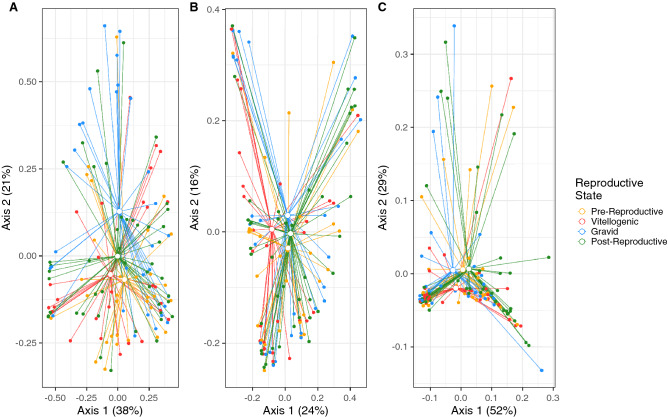
PCoA for cloacal microbiomes of female *S. virgatus* across four reproductive states, based on (**A**) Bray–Curtis, (**B**) unweighted UniFrac, and (**C**) weighted UniFrac distances. Open dots represent the centroids of each group, and solid dots represent each sample. Colors represent each reproductive state.

The female microbiome differed among reproductive states when examined using Bray–Curtis distance with a PERMANOVA (R^2^ = 0.04, p = 0.032), although there was no difference with weighted UniFrac distance (R^2^ = 0.03, p = 0.201), and only a trend of separation using unweighted UniFrac distance (R^2^ = 0.03, p = 0.070, Fig. 5, Table S9).

**Figure 6:**
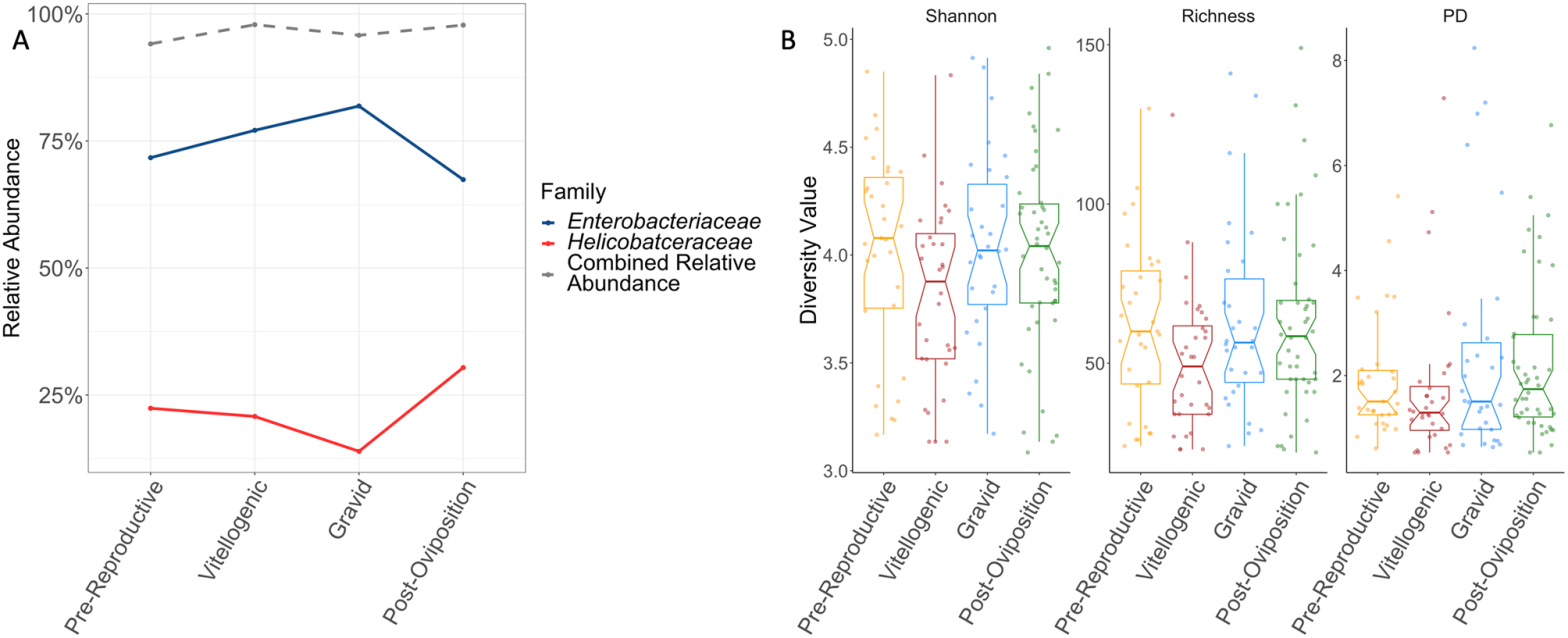
(**A**) Mean relative abundance of *Enterobacteriaceae* and *Helicobacteraceae* in cloacal microbiomes of female *S. virgatus* across four reproductive stages, as well as the total combined relative abundance of these two families. (**B**) Diversity (Shannon index), richness, and Faith’s phylogenetic diversity (PD) of cloacal microbiomes for female *S. virgatu*s across reproductive state. Boxes represent median and quartiles, while whiskers indicate 95% confidence intervals.

As described above, the female microbiome is largely dominated by *Enterobacteriaceae* across the entire active season. The microbiome of gravid females had the highest relative abundance of *Enterobacteriaceae* (81.8% ± 3.8), while post-reproductive females hosted the least (67.4% ± 0.49). Pre-reproductive and vitellogenic microbiomes contained 71.7% ± 4.4 and 77.1% ± 5.1, respectively, indicating a slight increase of the relative abundance of *Enterobacteriaceae* throughout the reproductive season, which then drops off after oviposition. The only other taxon that made up > 1.0% of the community on average, in any reproductive state, is *Helicobacteraceae*, which appears to vary in concert with *Enterobacteriaceae* (pre-reproductive: 22.4% ± 4.3; vitellogenic: 20.8% ± 4.9; gravid: 13.9% ± 3.9; post-oviposition: 30.4% ± 4.8, Fig. 6A). There was no significant variation in any alpha diversity metric across female reproductive states (ANOVA), although Shannon diversity and richness were consistently lower for vitellogenic females (Fig. 6B, Table S10).

## Discussion

By sampling wild *S. virgatus* over several years and seasons, we evaluated how the cloacal microbiome varies temporally and as a function of host-related factors. We found that the composition and diversity of the cloacal microbiomes of *S. virgatus* varied as a function of the period of the active season and the sex of the animal, while only composition varied as a function of body size. For females, we found limited but meaningful evidence for variation in composition by reproductive state. These factors all impact the microbiome independently of one another, as statistical models with interaction terms did not more effectively describe the variation we observed.

Despite differences at the ASV level, communities were remarkably consistent at deep taxonomic levels, with the combined average relative abundance of *Enterobacteriaceae* and *Helicobacteraceae* never falling below 93% for either sex in any season. High relative abundance of Proteobacteria is common in reptiles, although the values here were particularly high (see^6,25,41,42^), possibly reflecting special conditions in the *S. virgatus* cloaca.

The lack of a difference in the cloacal microbiome immediately before and after hibernation contrasts with studies of other species^43–45^. This suggests a stabilizing mechanism for the microbiome that plays a role even with the drastic environmental and metabolic shifts that occur during hibernation. This hypothetical mechanism also could explain consistency across years in cloacal microbiomes described above, and merits further study.

Even though abundance does not necessarily reflect functional significance, the resistance of core microbes to fluctuations, despite multiple overlapping influences, suggests the possibility of important function. Particularly at the family level, an extremely small number of taxa were consistent enough to be counted in a common core, as in other wild populations^46,47^. These prevalent taxa also tended to be the most abundant taxa in the community (most notably *Enterobacteriaceae*). The fact that the trends of the temporal core also matched the fluctuations across variables of the whole community supports the idea that these patterns are intrinsic to the population, and due to selection or adaptation. Previous research indicates that the cloacal microbiome serves to deposit microbes with antifungal capabilities on eggshells during oviposition^24^, and members of the *Enterobacteriaceae* have antifungal properties in this (unpublished data) and other systems^48–50^. However, we also note that some of the *Enterobacteriaceae* detected here (e.g., *Klebsiella*) represent genera that include pathogens of other reptiles^51,52^. *Helicobacteraceae* is the next most abundant taxon in the community, and certain *Helicobacteraceae* species are highly host-specific and have a long-term relationship with reptiles^53^. These lineages have been hypothesized to serve important functions in other lizard species^53^. These particular taxa are thus targets for functional analysis.

Although we have a broad picture of the taxa that are present in this community, the short sequences recovered via Illumina sequencing do not allow for confident taxonomic classification beyond the family or genus level. However, a similar dominance by *Enterobacteriaceae* was found in *S. virgatus* in a study using 16S rRNA clone libraries^8^. It will be valuable to sequence longer gene fragments of the bacteria in the community, both to identify more variable taxa, and to further explore the taxa that comprise the core microbiome and what their functional significance may be.

Even so, we examine these fine scale changes in the microbial community across the population and over time through the lens of ASVs. Female *S. virgatus* host more diverse microbiomes than males on average. This could be due to behavioral differences, as energy allocation, activity, and feeding habits differ between *S. virgatus* males and females^28,54^. Interestingly, the opposite pattern of sex difference was found in previous research on *S. virgatus*: in that study, microbial diversity of reproductive lizards (sampled in June) was higher in males than in females^8^. That previous work relied on clone libraries (25 clones/sample) and small sample sizes (n = 6 individuals/sex). Overall we consider the current data to be a more complete representation of the microbial community and a more reliable indication of true sex differences.

There are many examples of the microbiome varying by sex in vertebrates, including birds and mammals. Escallon et al.^7^ found that male rufous-collared sparrows had a more complex cloacal microbiome than females, but this difference only occurred in a specific season and is hypothesized to be the result of changes in testosterone levels. Variation in microbiome composition between sexes has also been found in mouse models and humans^55–58^, hypothesized to result from hormone variation rather than behavioral differences. Neither behavioral nor physiological mechanisms can be ruled out, but the fact that the sex difference persists year round, through multiple hormonal states and multiple behavioral transitions, suggests that the microbiome is resistant to these factors.

Other studies involving reptiles have found that males and females harbored similar microbiomes. For example, no sex differences were found by Kohl et al.^14^, who compared three liolaemid lizard species, nor by Montoya-Ciriaco et al.^15^, who examined *Sceloporus grammicus*. However, both of these studies used feces rather than cloacal swabs, which recover different communities^25,59^. Also, these studies examined viviparous lizards, which we expect to have different selective pressures than oviparous species like *S. virgatus*.

Differences in snout-vent length of *S. virgatus* was associated with shifts in the composition of the microbiome. This pattern has been well documented in other systems, generally with larger animals having more diverse microbiomes^60^. An increase in microbial diversity with body size has been linked to more gut space and longer transit time through the gut, giving microbes more time to colonize^61^. Female *S. virgatus* are larger than males, but the effect of size was independent of sex, and alpha diversity metrics remained constant, with only shifts in the overall community structure related to size. Size also roughly correlates with age in this species^62^, with adult females growing approximately 1.1 mm/yr^62^. Thus, animals in this study range from yearlings to approximately 8 years old, which is the longest lifespan known for any individual on our long-term study site. Microbiomes of other animals change as they age, particularly when shifting from juvenile to adult stages^3,7,9^, but this study focused only on adults.

We also found variation in the microbiome across different seasons of the active period. Diversity was lowest during the reproductive season, when females are developing and carrying eggs and intersexual social interactions peak^28,63^. However, this shift took place across the whole population, without an interaction of sex. Seasonal changes have been identified in both sexes of several other species, including mice^11^, alligators^12^, and other lizards^14^, all of which were hypothesized to be linked to seasonal shifts in diet. Seasonal changes in diet composition have not been described for *S. virgatus*, a generalist insectivore^64^. There has also been evidence in birds and lizards that sexual contact during mating can affect cloacal microbial composition^7,26^, and even non-sexual social contact can impact microbiomes of solitary mice^65^. Thus, the change in the *S. virgatus* microbiome as a function of the reproductive season could be due to an increase in both sexual contact and other social interactions, which occur during the mating season.

Our results contrast with most studies of microbiomes related to sociality: *S. virgatus* individuals have the lowest microbial diversity when they are at their most social. This also is reflected in the variation we found between the sexes, as males are generally more active than females^28^ but have lower diversity in their cloacal microbiomes. Lower diversity in association with increased sociality has been observed relatively rarely, but has been noted in lemurs^66^. Raulo et al*.*^66^ hypothesized that social interaction led to enrichment of already abundant taxa, rather than an increase in overall diversity. This seems like a possibility in this system as well, given the high abundance of *Enterobacteriaceae*. Additionally, some members of *Enterobacteriaceae* have antimicrobial properties^67–69^, which could further limit diversity if those particular taxa are enriched. We did not directly compare social interactions with the microbiome; we only related microbiome shifts to what is known about *S. virgatus* behavior within each timeframe. Changes observed here could reflect increased social interactions, but also could be due to physiological changes, including sex steroid and glucocorticoid hormones. Stress in particular has been shown to both result from certain social interactions and impact microbiome diversity^70–72^.

In addition to seasonal shifts in cloacal microbiomes, we identified some less consistent trends when examining only females across different reproductive states. On average, vitellogenic females (which are sexually receptive) tended to have lower alpha diversity in all three metrics, and diversity recovered during gravidity. Similar to changes in the whole population across activity periods, the lowest diversity here correlates with the period of highest social activity. There were significant differences across female reproductive states in the overall community when looking at beta diversity, with the major differences being among gravid females relative to pre-reproductive and vitellogenic females. The shift in community structure could be due to changes in food intake, as gravid females show a significant reduction in overall feeding frequency^73^. Remodeling of the gut microbiome during pregnancy occurs in other systems, but generally involves a simplification of the community^10,74^, opposed to the increase in diversity relative to vitellogenic animals seen here. Aside from being a side effect of behavioral changes, remodeling during gravidity could be a result of selective pressures, as evidence mounts that the maternal microbiome can be passed to offspring in reptiles and other oviparous animals^14,75,76^, as well serving important functions during egg incubation^24,77,78^.

## Conclusion

The cloacal microbiome of wild *S. virgatus* includes a resilient core microbiome that remains stable across years and through hibernation, but at a finer scale is impacted by the animal’s sex and body size, and the time point during the active season. Although there is evidence that the major taxa in this system have an important functional role, less is known about the functional impact of these small-scale changes, or mechanisms that may drive the changes. This is a rich area for future research. This study highlights the value of detailed, wide scale and long-term studies on wild populations: large datasets can allow researchers to parse changes in structure and function due to multiple overlapping factors. While it is tempting to apply patterns and trends from existing literature to new study species, even closely related species may have their own set of unique pressures that influence their resident microbes, such that caution should be used before making generalizations across species.

## Supplementary Information

Supplementary Information 1.

Supplementary Information 2.

Supplementary Information 3.

## Data Availability

The sequences and associated metadata used to support the conclusions of this study, including samples and controls, are available in the sequence read archive of NCBI (BioProject PRJNA813560). R scripts and csv files will be available on Dryad: https://doi.org/10.5061/dryad.qfttdz0k2.
