# Supplementary material for: Wild microbiomes of striped plateau lizards vary with reproductive season, sex, and body size
Source: Sci Rep. 2022 Nov 30;12:20643. doi: 10.1038/s41598-022-24518-6 (PMC9712514; doi:10.1038/s41598-022-24518-6)
Supplement: Supplementary file 1 — Supplementary Information 1. [file 41598_2022_24518_MOESM1_ESM.docx]

**Supplemental File 1: Wild microbiomes of striped plateau lizards vary with sex, body size, and reproductive season.**

**Marie E. Bunker^1^, A. Elizabeth Arnold^2^, and Stacey L. Weiss^1^**

**^1^**Department of Biology, University of Puget Sound, Tacoma, WA, USA

**^2^**School of Plant Sciences and Department of Ecology and Evolutionary Biology, The University of Arizona, Tucson, AZ, USA


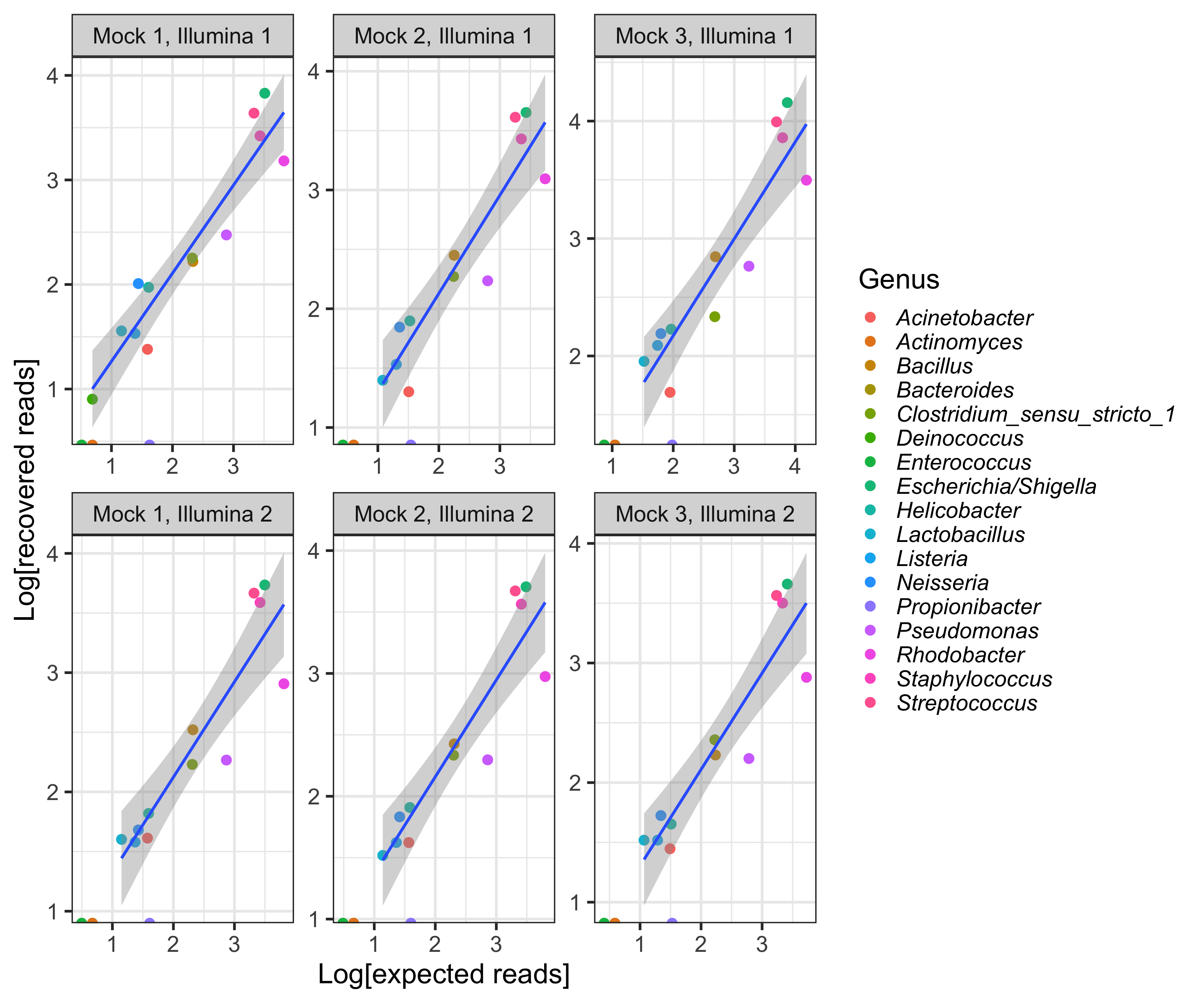


Figure S1. Expected reads compared to recovered reads of three mock community replicates used in two Illumina runs included in “Wild microbiomes of striped plateau lizards vary with sex, body size, and reproductive season,” using parameters described in the Methods section.


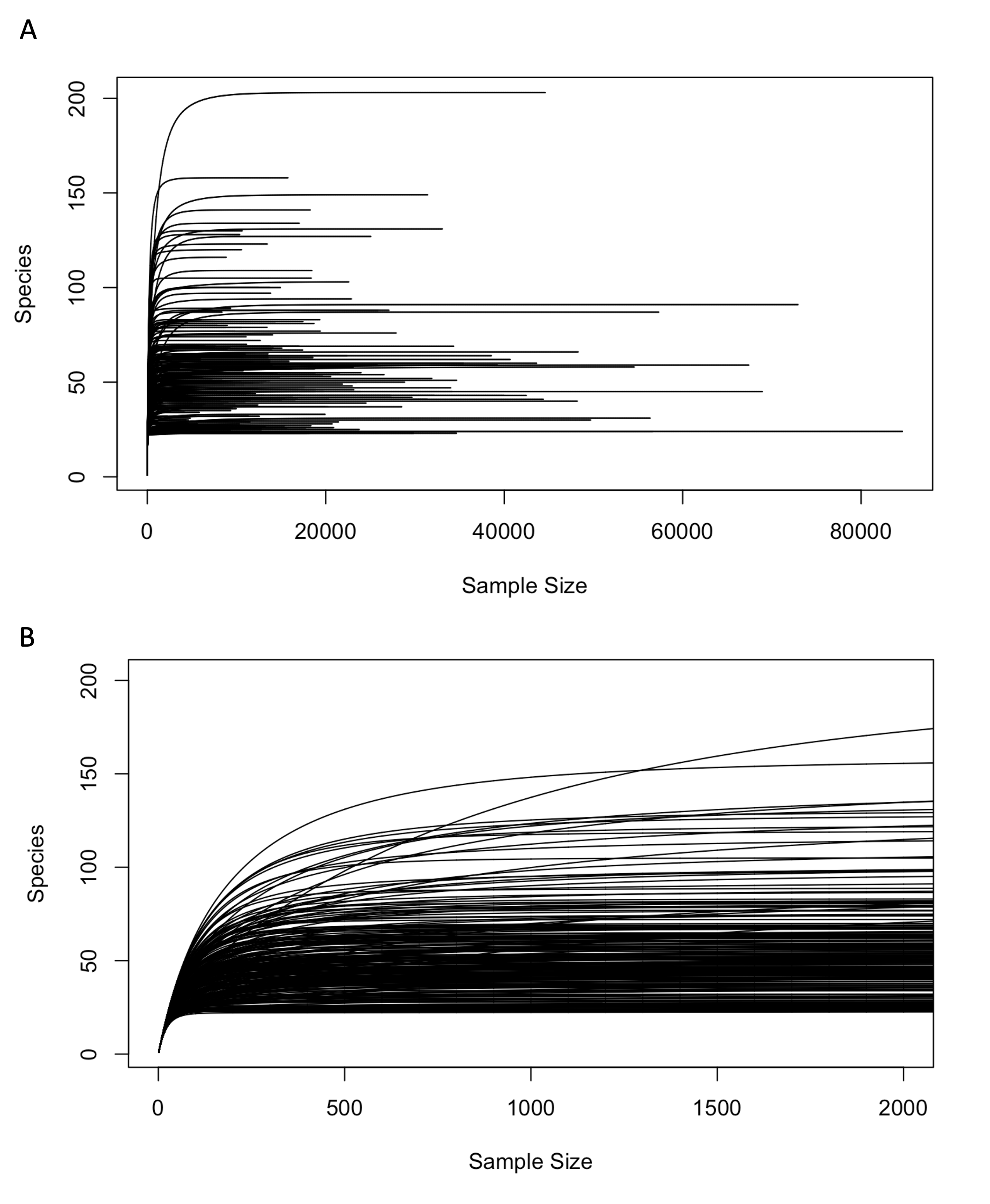


Figure S2. A. Rarefaction curves of recovered reads for all samples included “Wild microbiomes of striped plateau lizards vary with sex, body size, and reproductive season.” Read numbers plateau for all samples, indicating sequencing depth was sufficient to capture diversity. B. Same rarefaction curve zoomed into x-axis to more closely examine low-abundance sample curves.
