# Supplementary material for: Wild microbiomes of striped plateau lizards vary with reproductive season, sex, and body size
Source: Sci Rep. 2022 Nov 30;12:20643. doi: 10.1038/s41598-022-24518-6 (PMC9712514; doi:10.1038/s41598-022-24518-6)
Supplement: Supplementary file 2 — Supplementary Information 2. [file 41598_2022_24518_MOESM2_ESM.docx]

**Supplemental File 2: Justification of Approach to Detect and Remove Cloacal Swab Samples with Fecal Contamination**

**Marie E. Bunker^1^, A. Elizabeth Arnold^2^, and Stacey L. Weiss^1^**

**^1^**Department of Biology, University of Puget Sound, Tacoma, WA, USA

**^2^**School of Plant Sciences and Department of Ecology and Evolutionary Biology, The University of Arizona, Tucson, AZ, USA

**Methods**

Cloacal swabs were collected from female *Sceloporus virgatus* (n = 22) and processed as described in the main text. Swabs were visually inspected for signs of fecal contamination. Females were maintained in captivity in tanks sanitized with 70% ethanol, and fecal pellets were collected *ad libitum*. These samples – none of which are included in the analyses of the main text – were analyzed to assess potential fecal contamination of swab samples and to develop a bioinformatic approach to screening out such samples in other analyses.

**Results**

Communities vary between fecal pellets and swab samples, as well as between “contaminated” and “clean” cloacal swab communities (Figures S1 and S2).


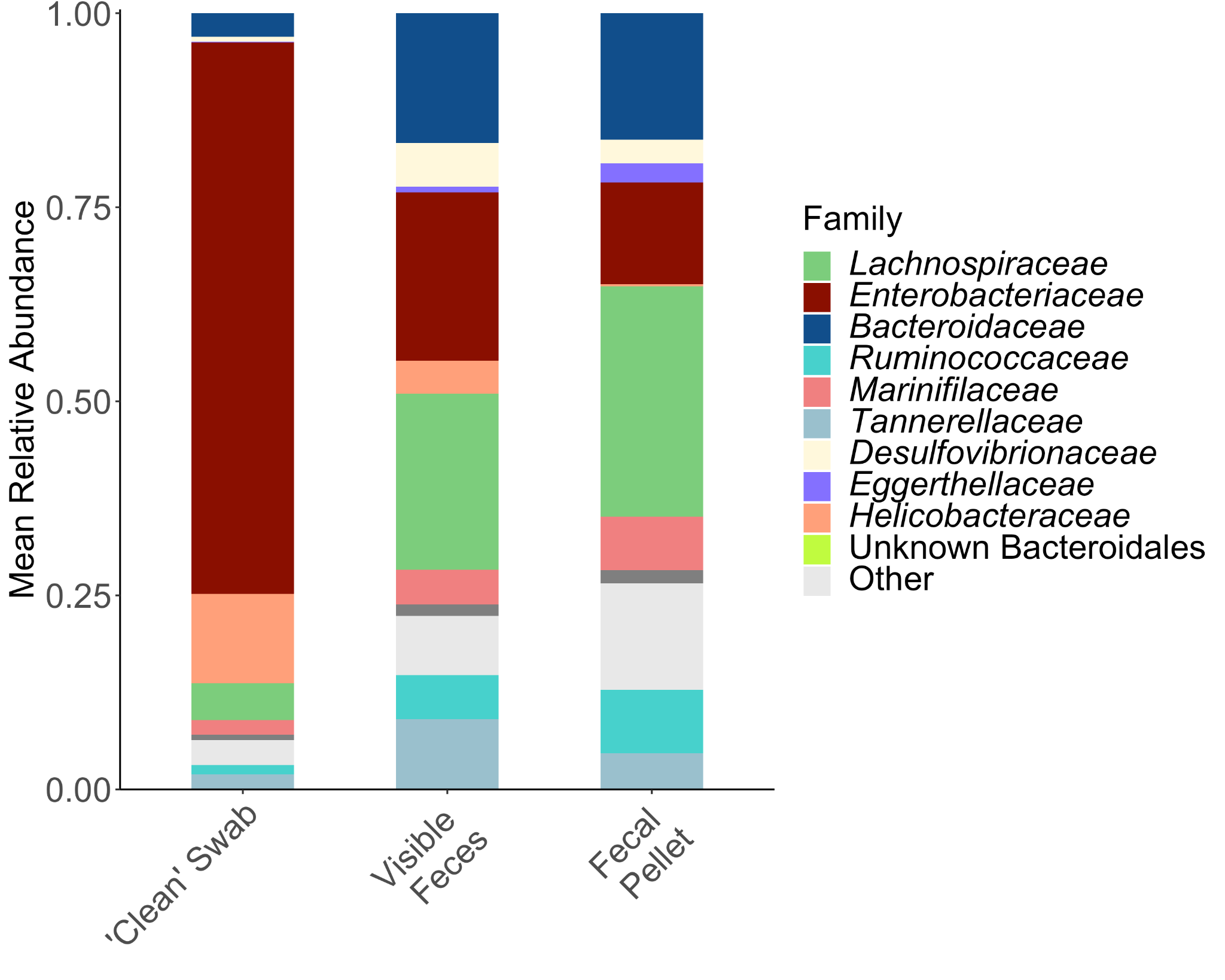


Figure S1. Mean relative abundance of top 10 most abundant families recovered from *S. virgatus* cloacal swabs with no visible contamination (“clean”), swabs with visible fecal contamination, and fecal pellets.


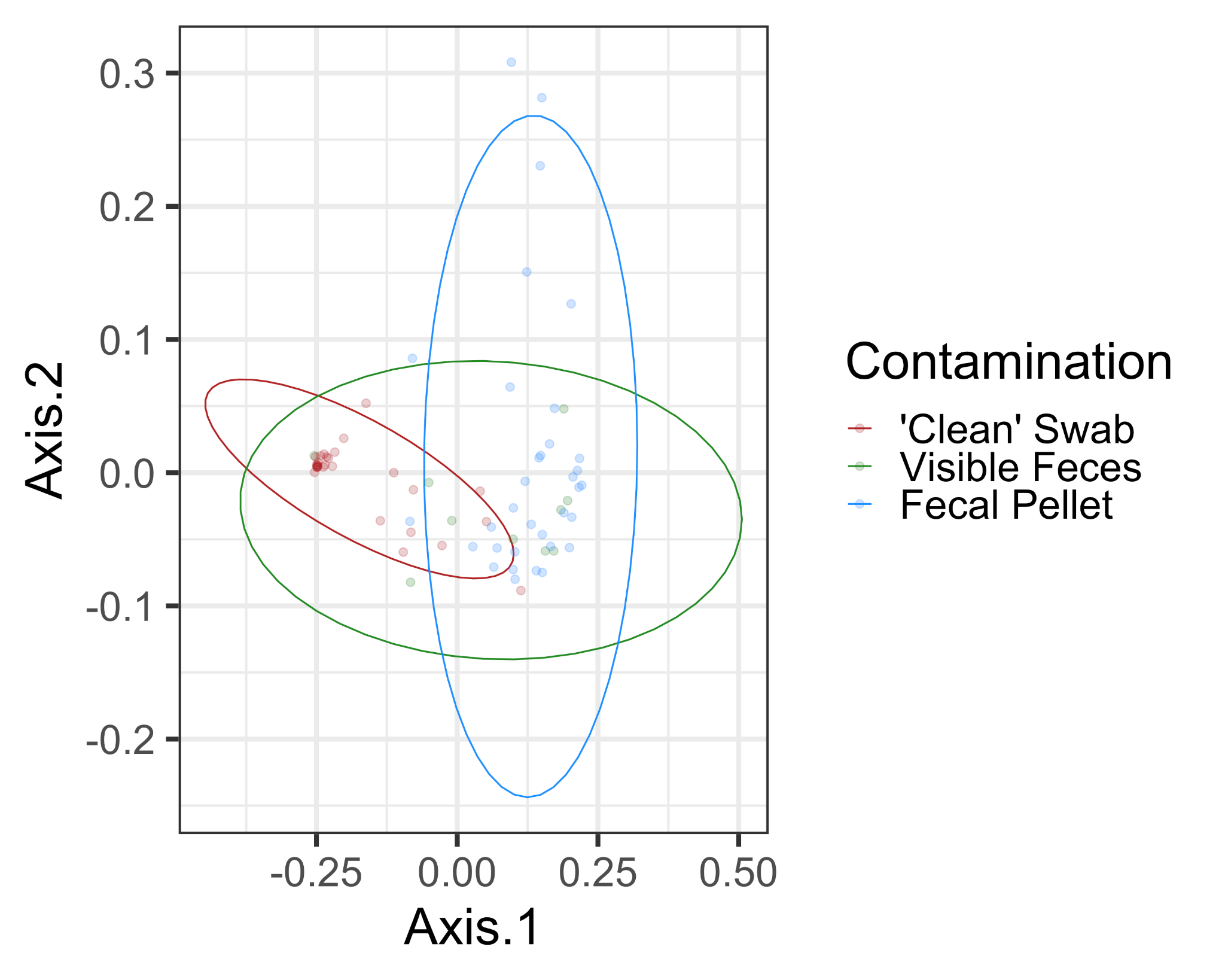


Figure S2. Community composition recovered from *S. virgatus* cloacal swabs with no visible contamination, swabs with visible fecal contamination, and fecal pellets.

Mean relative abundance of *Lachnospiraceae* is 29.7% ± 2.5 in fecal pellet samples, 22.7% ± 4.6 in visibly contaminated cloacal swabs, and 4.7% ± 1.4 in cloacal swabs with no visible feces. Permutational-ANOVA (PERMANOVA) confirms that the overall community varies between fecal pellets, contaminated swabs, and “clean” swabs (df = 2,72, R^2^ = 0.39, p = 0.001), as well as between contaminated and non-contaminated swabs only (df = 1,38, R^2^ = 0.29, p = 0.001). *Lachnospiraceae* is also differentially abundant between visibly contaminated and non-contaminated swabs (corncob analysis; t = 2.72, p = 0.01).

Removing samples with >5% relative abundance of *Lachnospiraceae* (roughly the average relative abundance in the “clean” swabs) removes all but 2 contaminated swabs and a single fecal pellet, while maintaining the most non-contaminated swabs possible (Fig S3). Some swabs with no note regarding fecal contamination were removed, but those communities indicate that small fecal particles likely adhered to the swab but were not visible or missed.


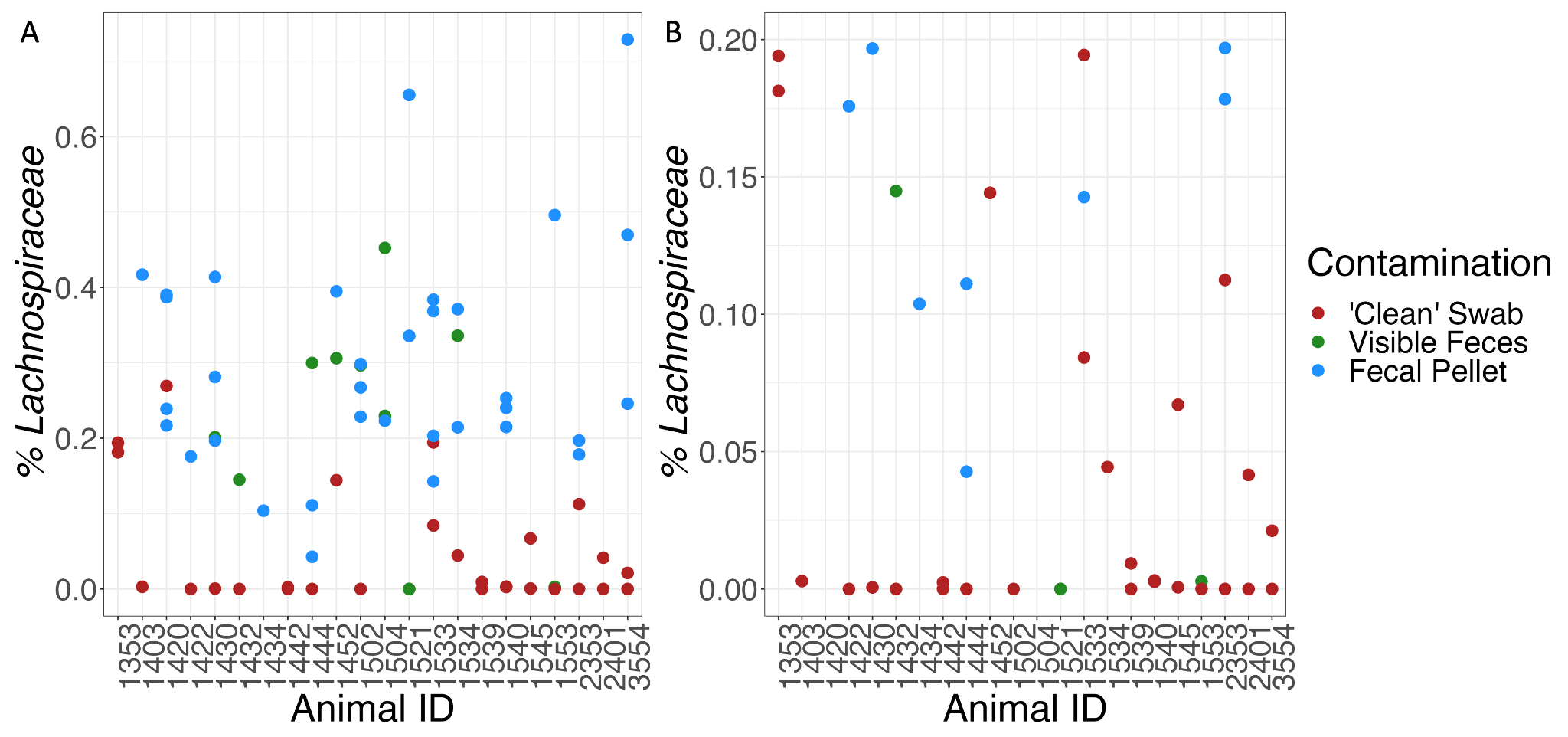


Figure S3. A. Relative abundance *Lachnospiraceae* in each sample. Points are colored by contamination status. B. Zoomed in x-axis to assess the 5% cutoff.
