# Supplementary material for: Wild microbiomes of striped plateau lizards vary with reproductive season, sex, and body size
Source: Sci Rep. 2022 Nov 30;12:20643. doi: 10.1038/s41598-022-24518-6 (PMC9712514; doi:10.1038/s41598-022-24518-6)
Supplement: Supplementary file 3 — Supplementary Information 3. [file 41598_2022_24518_MOESM3_ESM.docx]

**Supplemental Tables for: Wild microbiomes of striped plateau lizards vary with sex, body size, and reproductive season.**

**Marie E. Bunker^1^, A. Elizabeth Arnold^2^, and Stacey L. Weiss^1^**

**^1^**Department of Biology, University of Puget Sound, Tacoma, WA, USA

**^2^**School of Plant Sciences and Department of Ecology and Evolutionary Biology, The University of Arizona, Tucson, AZ, USA

Table S1: Statistical results for multivariate PERMANOVA model of the *S. virgatus* cloacal microbiome community in May-June as a function of year (2017, 2018, 2019), sex, and size (snout-vent length, svl). Bold font indicates a significant result

| **variable** | **DF** | **F value** | **P** | **variable** | **DF** | **F value** | **R^2^** | **P** |
| --- | --- | --- | --- | --- | --- | --- | --- | --- |
| *Betadispersion, Bray-Curtis Distance* | | | | *PERMANOVA, Bray-Curtis Distance* | | | | |
| year | 2 | 0.28 | 0.755 | year | 2 | 1.23 | 0.03 | 0.257 |
| sex | 1 | 6.37 | **0.014** | sex | 1 | 1.12 | 0.02 | 0.304 |
| svl | 20 | 2.12 | **0.012** | svl | 1 | 2.37 | 0.03 | **0.041** |
| *Betadispersion, Weighted UniFrac Distance* | | | | *PERMANOVA, Weighted UniFrac Distance* | | | | |
| year | 2 | 2.32 | 0.106 | year | 2 | 1.44 | 0.04 | 0.23 |
| sex | 1 | 1.43 | 0.236 | sex | 1 | 0.66 | 0.01 | 0.563 |
| svl | 20 | 1.23 | 0.266 | svl | 1 | 1.71 | 0.02 | 0.162 |
| *Betadispersion, Unweighted UniFrac Distance* | | | | *PERMANOVA, Unweighted UniFrac Distance* | | | | |
| year | 2 | 0.47 | 0.628 | year | 2 | 1.56 | 0.04 | 0.082 |
| sex | 1 | 0.21 | 0.65 | sex | 1 | 1.13 | 0.01 | 0.305 |
| svl | 20 | 1.97 | **0.024** | svl | 1 | 5.22 | 0.06 | **0.001** |

Table S2: Statistical results for three-way ANOVA of alpha diversity metrics of the *S. virgatus* cloacal microbiome as a function of year (2017, 2018, 2019), sex, and size (svl), with animal ID (toe.clip) included as a random factor.

| **variable** | **DF** | **F value** | **P** |
| --- | --- | --- | --- |
| Shannon ~ year + sex + svl + Error(toe.clip) | | | |
| Error: toe clip |  |  |  |
| year | 2 | 1.9 | 0.157 |
| sex | 1 | 2.65 | 0.108 |
| svl | 1 | 0.25 | 0.612 |
| log10(Richness) ~ year + sex + svl + Error(toe.clip) | | | |
| Error: toe clip |  |  |  |
| year | 2 | 1.98 | 0.146 |
| sex | 1 | 2.57 | 0.114 |
| svl | 1 | 0.31 | 0.582 |
| log10(PD) ~ year  + sex + svl + Error(toe.clip) | | | |
| Error: toe clip |  |  |  |
| year | 2 | 2.26 | 0.112 |
| sex | 1 | 1.22 | 0.273 |
| svl | 1 | 2.17 | 0.145 |

Table S3: Statistical results for multivariate PERMANOVA model of the *S. virgatus* cloacal microbiome community across the hibernation period (month), including the effect of sex and size (svl). Bold font indicates a significant result

| **variable** | **DF** | **F value** | **P** | **variable** | **DF** | **F value** | **R2** | **P** |
| --- | --- | --- | --- | --- | --- | --- | --- | --- |
| *Betadipsersion, Bray-Curtis Distance* | | | | *PERMANOVA, Bray-Curtis Distance* | | | | |
| month | 1 | 0.07 | 0.795 | month | 1 | 0.05 | 0.01 | 0.762 |
| sex | 1 | 2.87 | 0.096 | sex | 1 | 3.21 | 0.06 | **0.009** |
| svl | 20 | 0.28 | **0.018** | svl | 1 | 1.78 | 0.03 | 0.124 |
| *Betadipsersion, Weighted UniFrac Distance* | | | | *PERMANOVA, Weighted UniFrac Distance* | | | | |
| month | 1 | 0.24 | 0.625 | month | 1 | 0.49 | 0.01 | 0.688 |
| sex | 1 | 1.87 | 0.176 | sex | 1 | 6.06 | 0.10 | **0.006** |
| svl | 20 | 2.75 | **0.005** | svl | 1 | 4.67 | 0.07 | **0.011** |
| *Betadispersion, Unweighted UniFrac Distance* | | | | *PERMANOVA, Unweighted UniFrac Distance* | | | | |
| month | 1 | 1.18 | 0.282 | month | 1 | 1.00 | 0.02 | 0.387 |
| sex | 1 | 0.06 | 0.808 | sex | 1 | 1.31 | 0.02 | 0.203 |
| svl | 20 | 3.16 | **0.002** | svl | 1 | 2.05 | 0.04 | **0.026** |

Table S4: Statistical results for three-way ANOVA of alpha diversity metrics of the *S. virgatus* cloacal microbiome across the hibernation period, including the effect of sex and size (svl), with animal ID (toe.clip) included as a random factor. Bold font indicates a significant result

| **variable** | **DF** | **F value** | **P** |
| --- | --- | --- | --- |
| Shannon ~ month + sex + svl + Error(toe.clip) | | | |
| Error: toe clip |  |  |  |
| month | 1 | 0.45 | 0.505 |
| sex | 1 | 2.78 | 0.102 |
| svl | 1 | 9.46 | **0.004** |
| log10(Richness) ~ month + sex + svl + Error(toe.clip) | | | |
| Error: toe clip |  |  |  |
| month | 1 | 0.49 | 0.486 |
| sex | 1 | 2.65 | 0.110 |
| svl | 1 | 9.36 | **0.004** |
| log10(PD) ~ month  + sex + svl + Error(toe.clip) | | | |
| Error: toe clip |  |  |  |
| month | 1 | 0.116 | 0.735 |
| sex | 1 | 1.08 | 0.304 |
| svl | 1 | 8.87 | **0.005** |

Table S5. Statistical results for multivariate PERMANOVA model of the *S. virgatus* cloacal microbiome, including the effect of reproductive season, sex, and size (svl). Bold font indicates a significant result

| **variable** | **DF** | **F value** | **P** | **variable** | **DF** | **F value** | **R2** | **P** |
| --- | --- | --- | --- | --- | --- | --- | --- | --- |
| *Betadipsersion, Bray-Curtis Distance* | | | | *PERMANOVA, Bray-Curtis Distance* | | | | |
| season | 2 | 2.49 | 0.085 | season | 1 | 2.75 | 0.01 | **0.007** |
| sex | 1 | 19.96 | **< 0.001** | sex | 2 | 2.83 | 0.012 | **0.032** |
| svl | 20 | 4.08 | **< 0.001** | svl | 1 | 3.27 | 0.01 | **0.014** |
| *Betadipsersion, Weighted UniFrac Distance* | | | | *PERMANOVA, Weighted UniFrac Distance* | | | | |
| season | 2 | 1.63 | 0.199 | season | 1 | 2.11 | 0.01 | 0**.**071 |
| sex | 1 | 6.02 | **0.015** | sex | 2 | 3.12 | 0.02 | **0.034** |
| svl | 20 | 2.41 | **0.001** | svl | 1 | 2.93 | 0.01 | **0.044** |
| *Betadispersion, Unweighted UniFrac Distance* | | | | *PERMANOVA, Unweighted UniFrac Distance* | | | | |
| season | 2 | 1.02 | 0.360 | season | 1 | 2.42 | 0.01 | **0.002** |
| sex | 1 | 0.36 | 0.551 | sex | 2 | 2.93 | 0.02 | **0.006** |
| svl | 20 | 2.87 | **< 0.001** | svl | 1 | 5.08 | 0.02 | **0.001** |

Table S6: Statistical results for three-way ANOVA of alpha diversity metrics of the *S. virgatus* cloacal microbiome, including the effect of reproductive season, sex, and size (svl), with animal ID (toe.clip) included as a random factor. Bold font indicates a significant result

| **variable** | **DF** | **F value** | **P** |
| --- | --- | --- | --- |
| Shannon ~ season + sex + svl + Error(toe.clip) | | | |
| Error: toe clip |  |  |  |
| season | 2 | 3.712 | **0.026** |
| sex | 1 | 19.01 | **< 0.001** |
| svl | 1 | 0.49 | 0.484 |
| log10(Observed)~ season + sex + svl + Error(toe.clip) | | | |
| Error: toe clip |  |  |  |
| season | 2 | 3.79 | **0.024** |
| sex | 1 | 18.89 | **< 0.001** |
| svl | 1 | 0.52 | 0.472 |
| log10(PD)~ season+ sex + svl + Error(toe.clip) | | | |
| Error: toe clip |  |  |  |
| season | 2 | 1.98 | 0.141 |
| sex | 1 | 110.43 | **0.001** |
| svl | 1 | 1.53 | 0.218 |

| **Table S7: ASVs and associated taxa included in any calculated core microbiome. “X” indicates the ASVs is included in the core. “Rep” = reproductive** | | | | | | | | | | | | |
| --- | --- | --- | --- | --- | --- | --- | --- | --- | --- | --- | --- | --- |
| **ASV** | **Phylum** | **Class** | **Order** | **Family** | **Genus** | **Temporal Core** | **Common Core** | **Male Core** | **Female Core** | **Pre-Rep Core** | **Rep Core** | **Post-Rep Core** |
| ASV_164 | Actinobacteria | Actinobacteria | Corynebacteriales | Corynebacteriaceae | Corynebacterium | X |  |  |  |  |  |  |
| ASV_176 | Actinobacteria | Actinobacteria | Corynebacteriales | Corynebacteriaceae | Corynebacterium | X |  |  |  |  |  |  |
| ASV_1 | Proteobacteria | Gammaproteobacteria | Enterobacteriales | Enterobacteriaceae | NA | X |  |  | X |  |  |  |
| ASV_2 | Proteobacteria | Gammaproteobacteria | Enterobacteriales | Enterobacteriaceae | NA | X |  |  | X |  |  |  |
| ASV_3 | Proteobacteria | Gammaproteobacteria | Enterobacteriales | Enterobacteriaceae | NA | X |  |  | X |  |  | X |
| ASV_4 | Proteobacteria | Gammaproteobacteria | Enterobacteriales | Enterobacteriaceae | NA | X | X | X | X | X |  |  |
| ASV_5 | Proteobacteria | Gammaproteobacteria | Enterobacteriales | Enterobacteriaceae | NA | X |  |  | X |  |  | X |
| ASV_6 | Proteobacteria | Gammaproteobacteria | Enterobacteriales | Enterobacteriaceae | NA | X | X | X | X | X | X | X |
| ASV_8 | Proteobacteria | Gammaproteobacteria | Enterobacteriales | Enterobacteriaceae | NA | X |  |  | X |  |  | X |
| ASV_10 | Proteobacteria | Gammaproteobacteria | Enterobacteriales | Enterobacteriaceae | Izhakiella | X | X | X | X | X |  |  |
| ASV_11 | Proteobacteria | Gammaproteobacteria | Enterobacteriales | Enterobacteriaceae | NA | X |  |  | X |  |  | X |
| ASV_13 | Proteobacteria | Gammaproteobacteria | Enterobacteriales | Enterobacteriaceae | NA | X |  |  | X |  |  |  |
| ASV_14 | Proteobacteria | Gammaproteobacteria | Enterobacteriales | Enterobacteriaceae | NA | X |  |  | X |  |  |  |
| ASV_15 | Proteobacteria | Gammaproteobacteria | Enterobacteriales | Enterobacteriaceae | NA | X |  |  | X |  |  |  |
| ASV_16 | Proteobacteria | Gammaproteobacteria | Enterobacteriales | Enterobacteriaceae | NA | X |  |  | X |  |  |  |
| ASV_17 | Proteobacteria | Gammaproteobacteria | Enterobacteriales | Enterobacteriaceae | NA | X |  |  | X |  |  |  |
| ASV_18 | Proteobacteria | Gammaproteobacteria | Enterobacteriales | Enterobacteriaceae | Izhakiella | X | X | X | X | X |  |  |
| ASV_19 | Proteobacteria | Gammaproteobacteria | Enterobacteriales | Enterobacteriaceae | Izhakiella | X | X | X | X | X | X |  |
| ASV_22 | Proteobacteria | Gammaproteobacteria | Enterobacteriales | Enterobacteriaceae | Izhakiella | X | X | X | X | X |  |  |
| ASV_23 | Proteobacteria | Gammaproteobacteria | Enterobacteriales | Enterobacteriaceae | Izhakiella | X | X |  | X | X |  |  |
| ASV_25 | Proteobacteria | Gammaproteobacteria | Enterobacteriales | Enterobacteriaceae | NA | X |  |  | X |  |  |  |
| ASV_26 | Proteobacteria | Gammaproteobacteria | Enterobacteriales | Enterobacteriaceae | NA | X |  |  | X | X |  |  |
| ASV_27 | Proteobacteria | Gammaproteobacteria | Enterobacteriales | Enterobacteriaceae | NA | X |  |  | X |  |  |  |
| ASV_29 | Proteobacteria | Gammaproteobacteria | Enterobacteriales | Enterobacteriaceae | NA | X |  |  | X |  |  |  |
| ASV_31 | Proteobacteria | Gammaproteobacteria | Enterobacteriales | Enterobacteriaceae | Izhakiella | X |  |  | X | X |  |  |
| ASV_32 | Proteobacteria | Gammaproteobacteria | Enterobacteriales | Enterobacteriaceae | Izhakiella | X |  |  | X | X |  |  |
| ASV_34 | Proteobacteria | Gammaproteobacteria | Enterobacteriales | Enterobacteriaceae | Izhakiella | X |  |  | X | X |  |  |
| ASV_36 | Proteobacteria | Gammaproteobacteria | Enterobacteriales | Enterobacteriaceae | NA | X |  |  | X |  |  |  |
| ASV_37 | Proteobacteria | Gammaproteobacteria | Enterobacteriales | Enterobacteriaceae | Klebsiella | X |  |  | X |  |  |  |
| ASV_38 | Proteobacteria | Gammaproteobacteria | Enterobacteriales | Enterobacteriaceae | NA | X |  |  | X |  |  | X |
| ASV_40 | Proteobacteria | Gammaproteobacteria | Enterobacteriales | Enterobacteriaceae | NA | X |  |  | X |  |  |  |
| ASV_41 | Proteobacteria | Gammaproteobacteria | Enterobacteriales | Enterobacteriaceae | NA | X |  |  |  |  |  |  |
| ASV_42 | Proteobacteria | Gammaproteobacteria | Enterobacteriales | Enterobacteriaceae | NA | X |  |  |  |  |  |  |
| ASV_43 | Proteobacteria | Gammaproteobacteria | Enterobacteriales | Enterobacteriaceae | NA | X |  |  | X |  |  | X |
| ASV_44 | Proteobacteria | Gammaproteobacteria | Enterobacteriales | Enterobacteriaceae | NA | X |  |  | X |  |  |  |
| ASV_45 | Proteobacteria | Gammaproteobacteria | Enterobacteriales | Enterobacteriaceae | NA | X |  |  |  |  |  |  |
| ASV_46 | Proteobacteria | Gammaproteobacteria | Enterobacteriales | Enterobacteriaceae | Izhakiella | X |  |  | X | X |  |  |
| ASV_47 | Proteobacteria | Gammaproteobacteria | Enterobacteriales | Enterobacteriaceae | NA | X |  |  | X |  |  | X |
| ASV_48 | Proteobacteria | Gammaproteobacteria | Enterobacteriales | Enterobacteriaceae | Izhakiella | X |  |  | X | X |  |  |
| ASV_50 | Proteobacteria | Gammaproteobacteria | Enterobacteriales | Enterobacteriaceae | NA | X |  |  | X | X |  |  |
| ASV_51 | Proteobacteria | Gammaproteobacteria | Enterobacteriales | Enterobacteriaceae | NA | X |  |  |  |  |  |  |
| ASV_52 | Proteobacteria | Gammaproteobacteria | Enterobacteriales | Enterobacteriaceae | NA | X |  |  |  |  |  |  |
| ASV_55 | Proteobacteria | Gammaproteobacteria | Enterobacteriales | Enterobacteriaceae | Izhakiella | X |  |  | X | X |  |  |
| ASV_57 | Proteobacteria | Gammaproteobacteria | Enterobacteriales | Enterobacteriaceae | Izhakiella | X |  |  | X | X |  |  |
| ASV_58 | Proteobacteria | Gammaproteobacteria | Enterobacteriales | Enterobacteriaceae | NA | X |  |  |  |  |  |  |
| ASV_59 | Proteobacteria | Gammaproteobacteria | Enterobacteriales | Enterobacteriaceae | NA | X |  |  | X |  |  | X |
| ASV_60 | Proteobacteria | Gammaproteobacteria | Enterobacteriales | Enterobacteriaceae | NA | X |  |  |  |  |  |  |
| ASV_62 | Proteobacteria | Gammaproteobacteria | Enterobacteriales | Enterobacteriaceae | Salmonella | X |  |  |  |  |  |  |
| ASV_63 | Proteobacteria | Gammaproteobacteria | Enterobacteriales | Enterobacteriaceae | NA | X |  |  | X | X |  |  |
| ASV_64 | Proteobacteria | Gammaproteobacteria | Enterobacteriales | Enterobacteriaceae | Izhakiella | X |  |  | X | X |  |  |
| ASV_65 | Proteobacteria | Gammaproteobacteria | Enterobacteriales | Enterobacteriaceae | NA | X |  |  | X |  |  | X |
| ASV_69 | Proteobacteria | Gammaproteobacteria | Enterobacteriales | Enterobacteriaceae | NA | X |  |  | X |  |  |  |
| ASV_70 | Proteobacteria | Gammaproteobacteria | Enterobacteriales | Enterobacteriaceae | NA | X |  |  |  |  |  |  |
| ASV_71 | Proteobacteria | Gammaproteobacteria | Enterobacteriales | Enterobacteriaceae | NA | X |  |  | X |  |  | X |
| ASV_72 | Proteobacteria | Gammaproteobacteria | Enterobacteriales | Enterobacteriaceae | NA | X |  |  |  |  |  |  |
| ASV_73 | Proteobacteria | Gammaproteobacteria | Enterobacteriales | Enterobacteriaceae | NA | X |  |  | X |  |  |  |
| ASV_74 | Proteobacteria | Gammaproteobacteria | Enterobacteriales | Enterobacteriaceae | NA | X |  |  | X |  |  |  |
| ASV_75 | Proteobacteria | Gammaproteobacteria | Enterobacteriales | Enterobacteriaceae | NA | X |  |  |  |  |  |  |
| ASV_76 | Proteobacteria | Gammaproteobacteria | Enterobacteriales | Enterobacteriaceae | Salmonella | X |  |  |  |  |  |  |
| ASV_77 | Proteobacteria | Gammaproteobacteria | Enterobacteriales | Enterobacteriaceae | NA | X |  |  |  |  |  |  |
| ASV_78 | Proteobacteria | Gammaproteobacteria | Enterobacteriales | Enterobacteriaceae | NA | X |  |  | X |  |  |  |
| ASV_80 | Proteobacteria | Gammaproteobacteria | Enterobacteriales | Enterobacteriaceae | NA | X |  |  | X |  |  |  |
| ASV_81 | Proteobacteria | Gammaproteobacteria | Enterobacteriales | Enterobacteriaceae | NA | X |  |  | X |  |  |  |
| ASV_82 | Proteobacteria | Gammaproteobacteria | Enterobacteriales | Enterobacteriaceae | Salmonella | X |  |  |  |  |  |  |
| ASV_83 | Proteobacteria | Gammaproteobacteria | Enterobacteriales | Enterobacteriaceae | NA | X |  |  | X |  |  |  |
| ASV_85 | Proteobacteria | Gammaproteobacteria | Enterobacteriales | Enterobacteriaceae | NA | X |  |  |  |  |  |  |
| ASV_86 | Proteobacteria | Gammaproteobacteria | Enterobacteriales | Enterobacteriaceae | NA | X |  |  |  |  |  |  |
| ASV_88 | Proteobacteria | Gammaproteobacteria | Enterobacteriales | Enterobacteriaceae | NA | X |  |  |  |  |  |  |
| ASV_89 | Proteobacteria | Gammaproteobacteria | Enterobacteriales | Enterobacteriaceae | NA | X |  |  |  |  |  |  |
| ASV_91 | Proteobacteria | Gammaproteobacteria | Enterobacteriales | Enterobacteriaceae | Izhakiella | X |  |  |  |  |  |  |
| ASV_93 | Proteobacteria | Gammaproteobacteria | Enterobacteriales | Enterobacteriaceae | NA | X |  |  |  |  |  |  |
| ASV_94 | Proteobacteria | Gammaproteobacteria | Enterobacteriales | Enterobacteriaceae | NA | X |  |  |  |  |  |  |
| ASV_95 | Proteobacteria | Gammaproteobacteria | Enterobacteriales | Enterobacteriaceae | NA | X |  |  |  |  |  |  |
| ASV_96 | Proteobacteria | Gammaproteobacteria | Enterobacteriales | Enterobacteriaceae | NA | X |  |  |  |  |  |  |
| ASV_97 | Proteobacteria | Gammaproteobacteria | Enterobacteriales | Enterobacteriaceae | Izhakiella | X |  |  |  |  |  |  |
| ASV_98 | Proteobacteria | Gammaproteobacteria | Enterobacteriales | Enterobacteriaceae | NA | X |  |  |  |  |  |  |
| ASV_99 | Proteobacteria | Gammaproteobacteria | Enterobacteriales | Enterobacteriaceae | NA | X |  |  |  |  |  |  |
| ASV_100 | Proteobacteria | Gammaproteobacteria | Enterobacteriales | Enterobacteriaceae | NA | X |  |  |  |  |  |  |
| ASV_101 | Proteobacteria | Gammaproteobacteria | Enterobacteriales | Enterobacteriaceae | Serratia | X |  |  |  |  |  |  |
| ASV_103 | Proteobacteria | Gammaproteobacteria | Enterobacteriales | Enterobacteriaceae | NA | X |  |  |  |  |  |  |
| ASV_104 | Proteobacteria | Gammaproteobacteria | Enterobacteriales | Enterobacteriaceae | Salmonella | X |  |  |  |  |  |  |
| ASV_105 | Proteobacteria | Gammaproteobacteria | Enterobacteriales | Enterobacteriaceae | NA | X |  |  |  |  |  |  |
| ASV_106 | Proteobacteria | Gammaproteobacteria | Enterobacteriales | Enterobacteriaceae | NA | X |  |  |  |  |  |  |
| ASV_107 | Proteobacteria | Gammaproteobacteria | Enterobacteriales | Enterobacteriaceae | NA | X |  |  |  |  |  |  |
| ASV_108 | Proteobacteria | Gammaproteobacteria | Enterobacteriales | Enterobacteriaceae | NA | X |  |  |  |  |  |  |
| ASV_109 | Proteobacteria | Gammaproteobacteria | Enterobacteriales | Enterobacteriaceae | NA | X |  |  |  |  |  |  |
| ASV_110 | Proteobacteria | Gammaproteobacteria | Enterobacteriales | Enterobacteriaceae | Salmonella | X |  |  |  |  |  |  |
| ASV_111 | Proteobacteria | Gammaproteobacteria | Enterobacteriales | Enterobacteriaceae | Salmonella | X |  |  |  |  |  |  |
| ASV_112 | Proteobacteria | Gammaproteobacteria | Enterobacteriales | Enterobacteriaceae | NA | X |  |  |  |  |  |  |
| ASV_113 | Proteobacteria | Gammaproteobacteria | Enterobacteriales | Enterobacteriaceae | NA | X |  |  |  |  |  |  |
| ASV_114 | Proteobacteria | Gammaproteobacteria | Enterobacteriales | Enterobacteriaceae | NA | X |  |  |  |  |  |  |
| ASV_115 | Proteobacteria | Gammaproteobacteria | Enterobacteriales | Enterobacteriaceae | NA | X |  |  |  |  |  |  |
| ASV_116 | Proteobacteria | Gammaproteobacteria | Enterobacteriales | Enterobacteriaceae | NA | X |  |  |  |  |  |  |
| ASV_117 | Proteobacteria | Gammaproteobacteria | Enterobacteriales | Enterobacteriaceae | Serratia | X |  |  |  |  |  |  |
| ASV_118 | Proteobacteria | Gammaproteobacteria | Enterobacteriales | Enterobacteriaceae | NA | X |  |  |  |  |  |  |
| ASV_119 | Proteobacteria | Gammaproteobacteria | Enterobacteriales | Enterobacteriaceae | NA | X |  |  |  |  |  |  |
| ASV_120 | Proteobacteria | Gammaproteobacteria | Enterobacteriales | Enterobacteriaceae | NA | X |  |  |  |  |  |  |
| ASV_7 | Epsilonbacteraeota | Campylobacteria | Campylobacterales | Helicobacteraceae | Helicobacter | X | X | X | X | X |  | X |
| ASV_9 | Epsilonbacteraeota | Campylobacteria | Campylobacterales | Helicobacteraceae | Helicobacter | X |  | X | X | X |  | X |
| ASV_12 | Epsilonbacteraeota | Campylobacteria | Campylobacterales | Helicobacteraceae | Helicobacter | X | X | X | X | X |  | X |
| ASV_20 | Epsilonbacteraeota | Campylobacteria | Campylobacterales | Helicobacteraceae | Helicobacter | X |  | X | X | X |  |  |
| ASV_21 | Epsilonbacteraeota | Campylobacteria | Campylobacterales | Helicobacteraceae | Helicobacter | X |  | X | X | X |  | X |
| ASV_24 | Epsilonbacteraeota | Campylobacteria | Campylobacterales | Helicobacteraceae | Helicobacter | X |  | X | X | X |  |  |
| ASV_28 | Epsilonbacteraeota | Campylobacteria | Campylobacterales | Helicobacteraceae | Helicobacter | X |  | X | X | X |  |  |
| ASV_30 | Epsilonbacteraeota | Campylobacteria | Campylobacterales | Helicobacteraceae | Helicobacter | X |  | X | X | X |  |  |
| ASV_33 | Epsilonbacteraeota | Campylobacteria | Campylobacterales | Helicobacteraceae | Helicobacter | X |  | X | X | X |  |  |
| ASV_35 | Epsilonbacteraeota | Campylobacteria | Campylobacterales | Helicobacteraceae | Helicobacter | X |  | X | X | X |  | X |
| ASV_39 | Epsilonbacteraeota | Campylobacteria | Campylobacterales | Helicobacteraceae | Helicobacter | X |  | X | X | X |  |  |
| ASV_49 | Epsilonbacteraeota | Campylobacteria | Campylobacterales | Helicobacteraceae | Helicobacter | X |  | X | X | X |  |  |
| ASV_53 | Epsilonbacteraeota | Campylobacteria | Campylobacterales | Helicobacteraceae | Helicobacter | X |  | X | X | X |  |  |
| ASV_54 | Epsilonbacteraeota | Campylobacteria | Campylobacterales | Helicobacteraceae | Helicobacter | X |  | X | X | X |  |  |
| ASV_56 | Epsilonbacteraeota | Campylobacteria | Campylobacterales | Helicobacteraceae | Helicobacter | X |  | X | X |  |  |  |
| ASV_61 | Epsilonbacteraeota | Campylobacteria | Campylobacterales | Helicobacteraceae | Helicobacter | X |  | X | X |  |  |  |
| ASV_66 | Epsilonbacteraeota | Campylobacteria | Campylobacterales | Helicobacteraceae | Helicobacter | X |  | X | X | X |  |  |
| ASV_67 | Epsilonbacteraeota | Campylobacteria | Campylobacterales | Helicobacteraceae | Helicobacter | X |  | X | X |  |  |  |
| ASV_68 | Epsilonbacteraeota | Campylobacteria | Campylobacterales | Helicobacteraceae | Helicobacter | X |  | X | X |  |  |  |
| ASV_79 | Epsilonbacteraeota | Campylobacteria | Campylobacterales | Helicobacteraceae | Helicobacter | X |  |  |  |  |  |  |
| ASV_87 | Epsilonbacteraeota | Campylobacteria | Campylobacterales | Helicobacteraceae | Helicobacter | X |  |  |  |  |  |  |
| ASV_90 | Epsilonbacteraeota | Campylobacteria | Campylobacterales | Helicobacteraceae | Helicobacter | X |  |  |  |  |  |  |
| ASV_92 | Epsilonbacteraeota | Campylobacteria | Campylobacterales | Helicobacteraceae | Helicobacter | X |  |  |  |  |  |  |
| ASV_102 | Epsilonbacteraeota | Campylobacteria | Campylobacterales | Helicobacteraceae | Helicobacter | X |  |  |  |  |  |  |

Table S8. Statistical results for multivariate PERMANOVA model of the core microbial community of the *S. virgatus* cloacal microbiome, including the effect of reproductive season, sex, and size (svl), with animal ID (toe.clip) included as a random factor. Bold font indicates a significant result

| **variable** | **DF** | **F value** | **P** | **variable** | **DF** | **F value** | **R^2^** | **P** |
| --- | --- | --- | --- | --- | --- | --- | --- | --- |
| *Betadipsersion, Bray-Curtis Distance* | | | | *PERMANOVA, Bray-Curtis Distance* | | | | |
| season | 2 | 4.17 | 0.017* | season | 1 | 2.86 | 0.02 | **0.011** |
| sex | 1 | 20.86 | < 0.001* | sex | 2 | 3.11 | 0.01 | **0.032** |
| svl | 20 | 3.38 | < 0.001* | svl | 1 | 3.42 | 0.01 | **0.015** |
| *Betadipsersion, Weighted UniFrac Distance* | | | | *PERMANOVA, Weighted UniFrac Distance* | | | | |
| season | 2 | 0.87 | 0.421 | season | 1 | 1.4 | 0.01 | 0.264 |
| sex | 1 | 14.62 | < 0.001* | sex | 2 | 3.21 | 0.01 | 0.066 |
| svl | 20 | 2.29 | 0.002* | svl | 1 | 4.79 | 0.02 | **0.030** |
| *Betadispersion, Unweighted UniFrac Distance* | | | | *PERMANOVA, Unweighted UniFrac Distance* | | | | |
| season | 2 | 1.53 | 2.2 | season | 1 | 1.82 | **0.01** | **0.104** |
| sex | 1 | 6.06 | 0.014* | sex | 2 | 2.69 | 0.01 | **0.043** |
| svl | 20 | 2.25 | 0.002* | svl | 1 | 3.97 | 0.02 | **0.006** |

Table S9: Results of PERMANOVA model of the female *S. virgatus* cloacal microbiome in different reproductive states (RS). Bold font indicates a significant result

| **variable** | **DF** | **F value** | **P** | **variable** | **DF** | **F value** | **R^2^** | **P** |
| --- | --- | --- | --- | --- | --- | --- | --- | --- |
| *Betadipsersion, Bray-Curtis Distance* | | | | *PERMANOVA, Bray-Curtis Distance* | | | | |
| RS | 3 | 0.95 | 0.418 | RS | 3 | 1.85 | 0.04 | **0**.**032** |
| *Betadipsersion, Weighted UniFrac Distance* | | | | *PERMANOVA, Weighted UniFrac Distance* | | | | |
| RS | 3 | 1.33 | 0.266 | RS | 3 | 1.37 | 0.03 | 0.201 |
| *Betadispersion, Unweighted UniFrac Distance* | | | | *PERMANOVA, Unweighted UniFrac Distance* | | | | |
| RS | 3 | 1.39 | 0.247 | RS | 3 | 1.42 | 0.03 | 0.070 |

Table S10: Statistical results for one-way ANOVA of alpha diversity metrics of the female *S. virgatus* cloacal microbiome across reproductive states (RS), with animal ID (toe.clip) included as a random factor. Bold font indicates a significant result

| **variable** | **DF** | **F value** | **P** |
| --- | --- | --- | --- |
| Shannon ~ RS + Error(toe.clip) | | | |
| Error: toe clip |  |  |  |
| RS | 2 | 1.87 | 0.139 |
| log10(Richness) ~ RS + Error(toe.clip) | | | |
| Error: toe clip |  |  |  |
| RS | 2 | 1.89 | 0.136 |
| log10(PD) ~ RS + Error(toe.clip) | | | |
| Error: toe clip |  |  |  |
| RS | 2 | 1.61 | 0.191 |
